# Apical Sealing Ability of Mineral Trioxide Aggregate, Intermediate Restorative Material and Calcium Enriched Mixture Cement: A Bacterial Leakage Study

**DOI:** 10.22037/iej.2016.16

**Published:** 2016

**Authors:** Shahriar Shahriari, Farhad Faramarzi, Mohammad-Yousef Alikhani, Maryam Farhadian, Seyedeh Sareh Hendi

**Affiliations:** a *Department of Endodontics, Dental School, Hamedan University of Medical Science, Hamadan, Iran; *; b * Department of Microbiology, Medical School, Hamadan University of Medical Sciences, Hamadan, Iran; *; c * Modeling of Noncommunicable Diseases Research Center, Department of Biostatistics, School of Public Health, Hamadan University of Medical Sciences, Hamadan, Iran;*; d * Department of Endodontics, Dental School, Hamadan University of Medical Sciences, Hamadan, Iran*

**Keywords:** Apical Seal, Bacterial Leakage, Microleakage, Root-End Filling, Seal

## Abstract

**Introduction:**

This *in vitro *study compared the apical sealing ability of three common root end filling materials namely mineral trioxide aggregate (MTA), intermediate restorative material (IRM) and calcium-enriched mixture (CEM) cement using a bacterial leakage model.

**Methods and Materials:**

The study was conducted on 83 single-rooted human teeth. Tooth crowns were cut and root canals were prepared using the step-back technique. Apical 3 mm of the roots were cut and a three-mm-deep cavity was prepared using an ultrasonic instrument. The samples were divided into three groups (*n*=25) according to the root-end filling material including MTA, IRM and CEM cement. The roots were inserted into cut-end microtubes. After sterilization with ethylene oxide, microtubes were placed in sterile vials containing 10 mL of Brain Heart Infusion (BHI) broth and incubated at 37^°^C and 0.1 mL of *Enterococcus faecalis* suspension compatible with 0.5 McFarland standard (1.5×10^8 ^cell/ ml), which was refreshed daily. This procedure was continued for 70 days. The data were analyzed using the chi-square, Kruskal-Wallis and log rank tests. The level of significance was set at 0.05.

**Results:**

No significant difference was found in bacterial microleakage among three groups; MTA showed slightly (but not significantly) less microleakage than IRM and CEM. However, the difference in the mean time of microleakage was significant among the groups (*P*<0.04) and in MTA samples leakage occurred in a longer time than CEM (*P*<0.012).

**Conclusion:**

The three tested root end filling materials had equal sealing efficacy for preventing bacterial leakage.

## Introduction

In cases of endodontic failure, orthograde retreatment is commonly the first attempted approach. However, in case of failure of routine endodontic retreatment or when the orthograde approach is not feasible for any reason, endodontic surgery is often indicated to resolve apical periodontitis [1, 2]. The retrograde approach includes cutting and preparation of the root-end and filling the cavity with a root-end filling material to achieve an optimal apical seal [3]. Providing a good seal would protect periradicular tissues against microorganisms or their byproducts. Therefore, one of the most important factors playing a major role in the success of apical endodontic surgery is the type of sealing materials used [4].

Moreover, an ideal root end filling material should have dimensional stability, easy application and radiopacity and must be hydrophobic, insoluble in water and body fluids, non-toxic, non-carcinogenic, impermeable and biocompatible. Ideally, it must have antibacterial and antifungal properties and meanwhile it should not induce tooth discoloration [5]. Several materials have been recommended and used as an apical plug in retrograde endodontic surgery such as gutta-percha, amalgam, glass ionomer cement (GIC), intermediate restorative material (IRM), super EBA and *etc*. Gutta-percha contains a high level of zinc oxide which make it a toxic agent for living tissues [6]. Most of the aforementioned materials have many disadvantages that have forded researchers willing to always try to find alternatives [7]. It has been illustrated that GIC is not a suitable compound to be used in in endodontic surgery, because the compound would be damaged when it comes to contact with moisture [8]. Regarding this issue, there also are other alternatives with more pleasant properties, such as EBA and IRM and recently mineral trioxide aggregate (MTA) and calcium-enriched mixture (CEM) cement [9]. These compounds are able to promote the structural strength of zinc oxide-eugenol-based cements [10]. Moreover, previous studies have demonstrated that they cause no adverse effects on surrounding tissues. The Apical sealing ability of IRM has been shown to be acceptable by several studies [11, 12]. This compound is preferable from other aspects such as availability and causing no harm on surrounding tissues [13]. 

MTA is another compound which has been well accepted as the standard root-end filling material since it has excellent properties such as biocompatibility, non-toxicity, osteoinduction and cementogenesis and providing optimal apical seal [2, 14, 15]. However, MTA has some shortcomings such as long setting time, difficult handling, tooth discoloration potential and high cost [5]. To overcome these shortcomings, CEM cement, composed of calcium compounds, was later introduced [16], which has shown osteoinductivity and has the ability to set in aqueous environments. The film thickness and flow of CEM cement are significantly superior to those of MTA. Moreover, CEM cement has an antibacterial activity comparable to that of calcium hydroxide and higher than that of MTA. Also, CEM cement has a shorter setting and working time and is more affordable than MTA [1, 5, 17].

Milani *et al.* [18] evaluated the efficacy of MTA and CEM cement in obtaining seal in retrograde endodontic surgery and showed that the sealing ability of both materials was the same. Hasheminia *et al.* [3] assessed the sealing ability of MTA and CEM cement as root-end filling materials in blood-contaminated, saliva-contaminated and dry environments and indicated that CEM cement yielded superior results.

Considering the favorable properties of CEM cement and the importance of obtaining an optimal apical seal in retrograde endodontic surgery, this *in vitro* study compared the sealing ability of CEM cement, MTA and IRM using a bacterial leakage model. The null hypothesis was that the sealing ability of the three materials would not be significantly different.

## Materials and Methods

This *in vitro*, study was conducted on 83 human single-rooted teeth extracted for periodontal or orthodontic reasons. The inclusion criteria included absence of cracks or fracture, root caries or root resorption, no history of previous root canal therapy, a minimum of 10 mm of root length and initial apical file size being smaller than #30 K-file. The teeth were evaluated under a stereomicroscope to ensure absence of cracks or fracture. The sample size was calculated to be 25 samples in each of the three groups considering *α*=0.05, *β*=0.2 and study power of 80%. 

The teeth were immersed in 5.25% sodium hypochlorite solution for 1 h and were then stored in saline until the experiment. The crowns were cut by a diamond disc installed on a high-speed handpiece so that the remaining root length was 10±0.5 mm for the purpose of standardization. Actual root canal length was measured by inserting a K-file (Dentsply Maillefer, Ballaigues, Switzerland) into the canal until its tip was visible at the apex. Working length was determined 1 mm short of this length. Apical instrumentation of the roots was done up to #30 as the master apical file. After that, root canals were flared up to #50 using the step-back technique. During cleaning and shaping, root canal irrigation was performed using 5.25% sodium hypochlorite solution followed by a final rinse with saline. Three teeth were considered as the positive controls and their root ends were not filled with any filling material. Three teeth were considered as negative controls and were filled with wax to prevent microleakage. Next, apical 3 mm of the roots was cut at a 90^°^ angle relative to the longitudinal axis of the root by a diamond disc, and a three-mm-deep cavity was prepared by an ultrasonic instrument (Suprasson P5 Booster, France). A tapered gutta-percha cone was placed in the root canal 3 mm short of the prepared cavity. External root surfaces, except for the cut root end, were covered with two layers of nail varnish. The prepared roots were divided into three groups (*n*=25) for root-end filling with Angelus MTA (Angelus, Londrina, Paraná, Brazil), IRM (Caulk/Dentsply, Tulsa Dental, Tulsa, OK, USA) and CEM cement (Yektazist Dandan, Tehran, Iran). The root-end filling materials were prepared as recommended by the manufacturers and applied into the root-end cavities. Excess material was removed by a moist cotton pellet. To ensure the quality of fillings, radiographies were taken from all samples. Next, the teeth were incubated at 37^°^C and 100% humidity for 24 h. The dual chamber technique was used for assessment of bacterial leakage. The teeth were placed in cut-end microtubes (Artin company, Iran). The microtubes were cut at one end by 1.5 mm so that 3 to 5 mm of the root was left out of the microtube. The interface of tooth and microtube was sealed with sticky wax. Next, microtubes containing the teeth were sterilized with ethylene oxide for 12 h. The microtubes were then placed in sterile vials containing 10 cc of BHI (brain-heart infusion) broth (Merck, Darmstadt, Germany) under a hood in such a way that the opening of the vials perfectly fitted the microtubes while the roots were in the culture medium. To ensure the absence of contamination, the samples were incubated at 37^°^C for 24 h. No turbidity in the culture medium confirmed the accuracy of sterilization. 

*Enterococcus faecalis* (ATCC 29212) was obtained from the Microbiology Department of Hamadan University of Medical Sciences, Hamadan, Iran. To ensure purity, bacteria were cultured in blood agar media (Merck Co., Darmstadt, Germany). Pure bacterial colonies were cultured in 10 cc of BHI broth and incubated at 37^°^C for 24 h. The bacterial suspension was prepared compatible to 0.5 McFarland standard (1.5×10^8^) and 0.1 mL of the suspension was added to root canals on a daily basis. In case of occurrence of turbidity in the vials, its absorbance was read by a spectrophotometer (Gold Spectrumlab 53, BEL Photonic, FarazTajhizTeb, Tehran, Iran) at 600 nm wavelength. To ensure that the observed turbidity was due to proliferation of *E. faecalis*, specific tests using a special culture media were performed. The experiment was continued for 70 days. 

The data were analyzed using SPSS software (SPSS version 19.0, SPSS, Chicago, IL, USA) and the chi-square, Kruskal-Wallis and log rank tests were applied. To assess the significant differences in the time of bacterial leakage, survival analysis was performed based on the log rank test. The level of statistical significance was set at 0.05.

## Results

Tables 1 and 2, summarize the results of bacterial microleakage in experimental groups during the 70-day study period. Bacterial microleakage occurred in all positive control groups (100%) and in none of the negative control samples. The chi-square test showed no significant differences in bacterial microleakage among the three experimental groups (*P*=0.14). 

**Figure 1:**
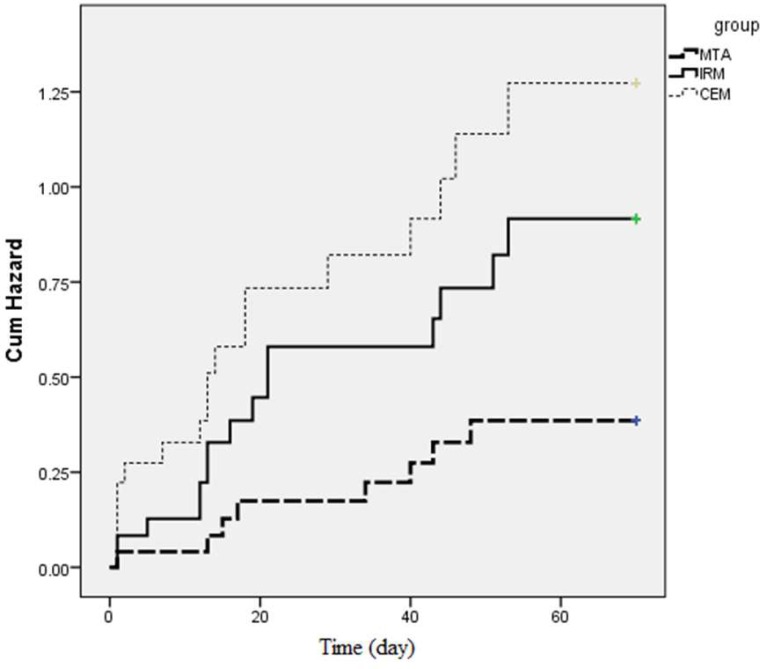
The Kaplan*-*Meier Survival curves for the occurrence of bacterial microleakage in the three experimental groups

Moreover, According to the log rank test, the difference in the meantime to microleakage among the experimental groups was statistically significant (*P*=0.04). Furthermore, Figure 1 shows the cumulative hazard plot of the occurrence of bacterial microleakage in the three experimental groups. As seen, MTA had the longest time to microleakage followed by IRM and CEM cement. Pairwise comparison of the groups by the log rank test showed that MTA had a significantly longer time to microleakage than CEM cement (*P*=0.012). However, no significant difference was found between MTA and IRM in time to microleakage (*P*=0.52). The difference in this respect between CEM cement and IRM was not significant either (*P*<0.06). Over time, the sealing ability of all three materials increased. As seen in Figure 1, the peak for the positive samples in the Y-axis decreased over time. 

## Discussion

Since the main goal of periapical surgery is to provide an ideal seal, selection of a root-end filling material with optimal sealability is extremely important in order to guarantee the success of endodontic surgery [2, 19, 20]. Several *in vitro* methods have been proposed for assessment of the sealing ability of apical restorative materials such as fluid filtration technique, bacterial leakage, radioisotope labeling, dye penetration method and electrochemical method. However, dye penetration and bacterial leakage models have been more commonly used [5, 18]. Dye penetration method is safe, affordable and accessible and penetration of dye can be quantitatively assessed; however, it only shows leakage in one plane (the mid-sagittal plane), and assessment of overall leakage with this method is not feasible. Moreover, the results obtained in this method have wide range of variability [2, 21]. For this reason, the bacterial leakage model was used in our study. However, the bacterial leakage method is qualitative and leakage of one type of bacteria can cause turbidity in the culture medium [5]. Accordingly, the high rate of false positive results and overestimation of leakage can be expected when using this technique*. *

**Table 1:** Frequency of bacterial microleakage in the experimental groups during study period (* the significance level of the Chi-square test

**Group (N)**	**Absence of microleakage N (%)**	**Presence of microleakage N (%)**	***P*** ** value**
**MTA (25)**	18 (72)	7 (28)	0.143^*^
**IRM (25)**	12 (48)	13 (52)	
**CEM (25)**	12 (48)	13 (52)	
**Total (25)**	42 (56)	33 (44)	

**Table 2:** Bacterial count in the three experimental groups (* the significance level of the Kruskal Wallis test

**Group (N)**	**Mean (SD)**	***P*** ** value**
**MTA (25)**	0.456 (0.680)	0.08*
**IRM (25)**	0.832 (0.698)	
**CEM (25)**	1.042 (0.665)	

**Table 3.** The mean time to bacterial microleakage in the three experimental groups (^* ^the significance level of the log rank test

**Group (N)**	**Mean time to microleakage**	**Minimum**	**Maximum**	***P*** **-value**
**MTA (25)**	58.333	50.489	66.177	0.04*
**IRM (25)**	44.478	34.096	54.861	
**CEM (25)**	39.950	28.085	51.150	
**Total (25)**	48.090	42.614	54.033	

*E. faecalis* was chosen for assessment of bacterial microleakage since it is the dominant strain responsible for chronic apical periodontitis secondary to a failed endodontic treatment. The duration of this experiment was 70 days according to the study by Kazem *et al.* [2] that compared the microleakage of CEM cement, Root MTA, White MTA and amalgam using dye penetration and bacterial leakage methods and reported that in both methods, CEM cement showed higher leakage than MTA, but this difference did not reach statistical significance [2]. The results of the current study showed that the percentage of samples with leakage was 28% and 52% for MTA and IRM/CEM, respectively. However, this difference was not statistically significant. Our study was similar regarding the method of assessment (bacterial leakage model) and duration of experiment (70 days). However, the sample size in the present assessment was double the value in the aforementioned study. Both studies showed that CEM cement is a suitable alternative to MTA as a root-end filling material. 

Asgary *et al.* [22] assessed the sealing ability of MTA, IRM and CEM cement using dye penetration method and showed that CEM cement had the highest sealing ability followed by MTA and IRM. In their study, similar to ours, CEM cement and MTA were not significantly different in terms of microleakage; however, in our study IRM showed similar sealing ability to that of CEM cement, but lower than that of MTA (although not significantly) while in their study IRM had significantly lower sealing ability than both MTA and CEM cement. Torabinejad *et al.* [23] also used the dye penetration model to assess the sealing ability of MTA, IRM and amalgam and indicated that MTA had a better sealability than IRM and amalgam. Hasheminia *et al.* [3] assessed the sealing ability of MTA and CEM cement in saliva-contaminated, blood-contaminated and dry environments and showed that the sealing ability of MTA and CEM cement was not significantly different in dry and blood-contaminated environments; however, CEM cement showed a better sealing ability than MTA in presence of saliva. This controversy in the results may be due to the presence of several types of bacteria in the saliva; while in our study, only one type of bacteria was assessed. Also, the methodology of the two studies was different. Yavari *et al.* [24] compared the microleakage of CEM cement, MTA, amalgam and composite as intra-orifice barriers using bacterial leakage model and saliva and demonstrated that CEM cement and MTA were not significantly different but caused less microleakage than amalgam and composite resin. Their methodology was similar to the present study; the only exception was that they used saliva instead of *E. faecalis* for bacterial contamination. Their results, also confirmed the absence of a significant difference between MTA and CEM in terms of sealing ability. These findings show that the contaminating agent probably plays no role in the obtained results. In contrast, Hasheminia *et al*. [3] showed that CEM cement provided a better seal in saliva-contaminated environment compared to MTA; such a difference in the results may be due to different methodology of the studies. 

In general, both MTA and CEM provide an optimal apical seal, which is attributed to their hydrophilic nature, good antibacterial/antifungal activity, high pH and the formation of hydroxyapatite crystals [24]. The MTA powder contains small hydrophilic particles that set in presence of moisture, thus, its application in the periapical area (where moisture control is difficult) does not compromise the quality of filling. Its pH remains high (12.5) for long periods of time and results in the release of calcium ion and formation of calcium hydroxide [25]. Also, MTA has shown optimal biological properties since it results in formation of mineralized tissue in close contact with the filling material, less apical inflammation and deposition of cementum in comparison with amalgam, Super EBA, IRM and ZOE, which have none of these characteristics [26]. 

The application of CEM cement is also easy since it is not sticky after mixing and does not adhere to the applicator. Therefore, it can be well adapted to the cavity walls. Since it is a water-based cement, moisture has no adverse effect on it. In humid environments, it releases high amounts of hydroxyl, calcium and phosphate ions that increase pH and result in formation of hydroxyapatite and also confer antibacterial activity [3]. 

Over time, the bacterial leakage of all three materials decreased, which may be due to the expansion of MTA and CEM cement over time. Although the current study showed equal sealing ability of CEM cement and MTA, this study had the limitations of in vitro conditions. Thus, generalizability of the results to the clinical setting must be done with caution. Future studies are required in the clinical setting to better elucidate this topic.

## Conclusion

CEM cement can be successfully used as an alternative to the commonly used root end filling materials such as MTA and IRM.
